# ^18^F-Mefway PET Imaging of Serotonin 1A Receptors in Humans: A Comparison with ^18^F-FCWAY

**DOI:** 10.1371/journal.pone.0121342

**Published:** 2015-04-01

**Authors:** Jae Yong Choi, Chul Hyoung Lyoo, Jin Su Kim, Kyeong Min Kim, Jee Hae Kang, Soo-Hee Choi, Jae-Jin Kim, Young Hoon Ryu

**Affiliations:** 1 Department of Nuclear Medicine, Gangnam Severance Hospital, Yonsei University College of Medicine, Seoul, Korea; 2 Department of Neurology, Gangnam Severance Hospital, Yonsei University College of Medicine, Seoul, Korea; 3 Molecular Imaging Research Center, Korea Institute of Radiological and Medical Sciences, Seoul, Korea; 4 Department of Chemistry and Biochemistry, Swarthmore College, 500 College Avenue Swarthmore, PA, United States of America; 5 Department of Psychiatry and Institute of Human Behavioral Sciences, Seoul National University College of Medicine, Seoul, Korea; 6 Department of Psychiatry and Institute of Behavioral Science in Medicine, Yonsei University College of Medicine, Seoul, Korea; Wayne State University, UNITED STATES

## Abstract

**Introduction:**

The purpose of this research is to evaluate the prospects for the use of 4-(*trans*-^18^F-fluoranylmethyl)-*N*-[2-[4-(2-methoxyphenyl)piperazin-1-yl]ethyl]-*N*-pyridin-2-ylcyclohexane-1-carboxamide (^18^F-Mefway) in comparison to ^18^F-*trans*-4-fluoro-*N*-2-[4-(2-methoxyphenyl)piperazin-1-yl]ethyl]-*N*-(2-pyridyl)cyclohexanecarboxamide (^18^F-FCWAY) for the quantification of 5-HT_1A_ receptors in human subjects.

**Method:**

Five healthy male controls were included for two positron emission tomography (PET) studies: ^18^F-FCWAY PET after the pretreatment with 500 mg of disulfiram and two months later, ^18^F-Mefway PET without disulfiram. Regional time-activity curves (TACs) were extracted from nine cortical and subcortical regions in dynamic PET images. Using cerebellar cortex without vermis as reference tissue, in vivo kinetics for both radioligands were compared based on the distribution volume ratio (DVR) calculated by non-invasive Logan graphical analysis and area under the curve ratio of the TACs (AUC ratio).

**Result:**

Although the pattern of regional uptakes in the ^18^F-Mefway PET was similar to that of the ^18^F-FCWAY PET (highest in the hippocampus and lowest in the cerebellar cortex), the amount of regional uptake in ^18^F-Mefway PET was almost half of that in ^18^F-FCWAY PET. The skull uptake in ^18^F-Mefway PET was only 25% of that in ^18^F-FCWAY PET with disulfiram pretreatment. The regional DVR values and AUC ratio values for ^18^F-Mefway were 17—40% lower than those of ^18^F-FCWAY. In contrast to a small overestimation of DVR values by AUC ratio values (< 10%) in ^18^F-FCWAY PET, the overestimation bias of AUC ratio values was much higher (up to 21%) in ^18^F-Mefway PET.

**Conclusion:**

As ^18^F-Mefway showed lower DVR values and greater overestimation bias of AUC ratio values, ^18^F-Mefway may appear less favorable than ^18^F-FCWAY. However, in contrast to ^18^F-FCWAY, the resistance to *in vivo* defluorination of ^18^F-Mefway obviates the need for the use of a defluorination inhibitor. Thus, ^18^F-Mefway may be a good candidate PET radioligand for 5-HT_1A_ receptor imaging in human.

## Introduction

Serotonin 1A (5-hydroxytryptamine, 5-HT_1A_) receptors in the central nervous system belong to the G-protein coupled receptor family and play an important role in cognitive and emotional functions. Growing evidence demonstrates that the changes in 5-HT_1A_ receptor binding has been strongly implicated in the etiology of mental illnesses such as depression [1,2], anxiety [3,4] and schizophrenia [5]. Thereby, the necessity of in vivo molecular imaging markers for 5-HT_1A_ receptors has been increasingly recognized [6,7].

Many radioligands for positron emission tomography (PET) imaging of 5-HT_1A_ receptors have been developed, the majority of which are structural analogues of *N*-[2-[4-(2-methoxyphenyl)-1-piperazinyl]ethyl]-*N*-2-pyridinylcyclohexanecarboxamide (WAY-100635), a 5-HT_1A_ receptor antagonist with a highly selectivity and affinity [8]. Although extensive investigation has been performed to find effective PET radioligands in the last decades, only three radioligands are being used studies in human subjects: [^11^C]WAY-100635, ^18^F-*trans*-4-fluoro-*N*-2-[4-(2-methoxyphenyl)piperazin-1-yl]ethyl]-*N*-(2-pyridyl)cyclohexanecarboxamide (^18^F-FCWAY), and 4-^18^F-fluoranyl-*N*-[2-[4-(2-methoxyphenyl)piperazin-1-yl]ethyl]-*N*-pyridin-2-ylbenzamide (^18^F-MPPF).

The ^11^C-based radioligands have an intrinsic limitation for clinical studies because their short half-life of 20 min requires a expensive cyclotron in the proximity of PET imaging facilities. Besides these currently available ^11^C-labelled radioligands, ^18^F-FCWAY showed considerable defluorination in vivo [9,10] and ^18^F-MPPF was susceptible to P-glycoprotein (P-gp) [11,12]. In vivo defluorination hampers the exact quantification of 5-HT_1A_ receptor binding in superficial brain areas due to contamination from radioactivity in the skull. This defluorination in humans can be ameliorated by the pre-administration of disulfiram, resulting in improved brain PET images. However, as the disulfiram is difficult to use in practice and potentially neurotoxic [13,14], there are limitations in widespread use of disulfiram for clinical study of ^18^F-FCWAY PET.

**Fig 1:**
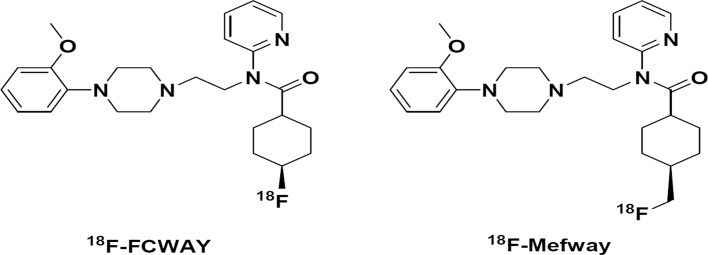
Chemical structures of ^18^F-FCWAY and ^18^F-Mefway.

To overcome this defluorination problem, the Mukherjee group developed 4-(*trans*-^18^F-fluoranylmethyl)-*N*-[2-[4-(2-methoxyphenyl)piperazin-1-yl]ethyl]-*N*-pyridin-2-ylcyclohexane-1-carboxamide (^18^F-Mefway). This radiotracer extends the carbon bond on the cyclohexyl ring of ^18^F-FCWAY to strengthen metabolic stability in vivo (Fig. 1). ^18^F-Mefway showed high affinity to the 5-HT_1A_ receptors, excellent target-to-reference ratio, high-quality 5-HT_1A_ specific images in the rhesus monkey even without disulfiram and favorable characteristics in human subject including low test-retest variability[15–18]. Moreover, ^18^F-Mefway showed kinetic properties, whole-body biodistribution, and dosimetry similar to [carbonyl-^11^C]WAY-100635[17]. Thus, in the present study we evaluated ^18^F-Mefway for the quantification of 5-HT_1A_ receptors in comparison with ^18^F-FCWAY in human subjects.

## Material and Methods

### Ethic clearance

Five healthy male subjects (age = 27.6 ± 2.2 years) participated in this study. Participants were screened using self-report questionnaires, including the Social Interaction Anxiety Scale [19],Social Phobia Scale [19], Brief Version of the Fear of Negative Evaluation Scale [20], and Beck Depression Inventory [21]. All participants were medication-naïve and had no history of psychiatric disorder. Institutional Review Board of Gangnam Severance Hospital ethically approved this study. The individual in this study has given written informed consent.

### Radiosynthesis

^18^F-FCWAY and ^18^F-Mefway were prepared as previously described [22,23]. Specific activities at the time of injection were 103.2 ± 38.3 and 117.8 ± 20.5 GBq/μmol, respectively. The radiochemical purities of these radioligands were greater than 99%.

### Acquisition of images

All five subjects underwent two PET scans: first with ^18^F-FCWAY and second with ^18^F-Mefway. For ^18^F-FCWAY PET, 500 mg of oral disulfiram was administered at 24 hours before the PET scan to inhibit in vivo defluorination. Participants were instructed not to drink alcohol the day before and 14 days after taking disulfiram [24]. After two months, ^18^F-Mefway PET scans were performed without pretreatment with disulfiram. All brain PET images were acquired with an integrated PET with computed tomography (PET/CT) scanner (Biograph 40 TruePoint PET/CT, Siemens Healthcare, Knoxville, TN, USA). A head holder was applied to minimize head movement, and brain CT images were acquired for attenuation correction (120 KeV, 180 mAs, 3 mm of slice thickness, 512 x 512 x 110 matrix with 0.67 x 0.67 x 2 mm of voxel size). After the bolus injection of radiotracers for 1 minute (259.7 ± 23.7 MBq for ^18^F-FCWAY and 276.8 ± 29.2 MBq for ^18^F-Mefway), we acquired dynamic PET scans data for 2 hours. Then, 33 time frames (6 x 30 sec, 3 x 1 min, 2 x 2 min, and 22 x 5 min) of attenuation-corrected dynamic PET images were reconstructed in the same matrix and with same voxel size as the CT images by using the ordered-subsets expectation maximization (OSEM) algorithm (iteration = 6 and subset = 16). In all subjects, high resolution T1-weighted brain magnetic resonance (MR) images were acquired in 1.5 T MR scanner (Signa Horizon, GE Medical Systems, Milwaukee, WI, USA) by using a fast spoiled gradient echo sequence (matrix = 256 x 256 x 116 matrix, voxel size = 0.94 x 0.94 x 1.5 mm, repetition time = 8.5 ms, echo time = 1.8 ms, field of view = 240 mm, flip angle = 12^o^).

### Preprocessing of PET images

For motion correction, dynamic PET images were realigned to mean PET images except first three time frames by sum of squared difference dissimilarity measure and Powell algorithm (PMOD 3.1 software, PMOD Technologies Ltd., Adliswil, Switzerland). T1-weighed MR images were corrected for inhomogeneity and segmented into gray matter, white matter and cerebrospinal fluid with statistical parametric mapping 8 (SPM8; The Wellcome Trust Centre for Neuroimaging, University College London, UK) implemented in MATLAB 8 (MathWorks, Natick, MA). The segments of gray and white matter were binarized with a threshold 0.5 to create whole brain mask, and skull-stripped MR images were created by overlaying the whole brain mask on inhomogeneity-corrected MR images. Mean PET images were coregistered to the skull-stripped MR images. The dynamic PET images were spatially normalized to template space by using the transformation parameter normalizing skull stripped MR images to the skull-stripped Montreal Neurologic Institute (MNI) MR template. Finally, time-activity curves (TACs) of seven cortical (frontal, parietal, occipital, temporal, insula, cingulate cortices, and hippocampus) and two subcortical (striatum and cerebellum) regions were obtained by overlaying template volumes of interest (VOI) modified from automated anatomical labeling (AAL) template. For the cerebellum, the vermis was excluded from VOI since this area contains small amount of 5-HT_1A_ receptors [25]. The accumulation of radioactivity in skull caused by the partial-volume effect was evaluated. To obtain exact TAC of skull, individual cranial bone was extracted from CT image, and this mask for cranial bone was overlaid on individual PET images. Regional TACs were normalized for the body weight and administrated dose and converted to standardized uptake value (SUV) (radioactivity in kBq/cc x body weight in kg/injected dose in MBq).

### Kinetic analysis for binding values

We used cerebellar cortex without cerebellar vermis as reference tissue to obtain regional binding values. By using Ichise’s multilinear reference tissue model (MRTM), individual *k*_2_’ was obtained from TAC of combined region (hippocampus and insula) with fixed time for the linearization (t*) at 60 minute [26]. Then with these parameters, regional distribution volume ratio (DVR) values were calculated by using non-invasive Logan’s graphical analysis method [27]. Parametric DVR images were created for visualization with same parameters and model.

Using 60 to 100 minute data, we also calculated regional area under the curve of TAC (AUC) ratio (AUC_target_ / AUC_reference_) with trapezoid method. Because the target-to-reference ratios show the plateau in this period.

### Statistical analysis

The Mann-Whitney U test was used to compare the DVR values between two radiotracers. Pearson's correlation was used to determination of relationship between DVR and AUC ratio. All statistical analyses were performed with Prism 5 (ver. 5.04, GraphPad).

## Results

### Comparison of skull uptake

**Fig 2:**
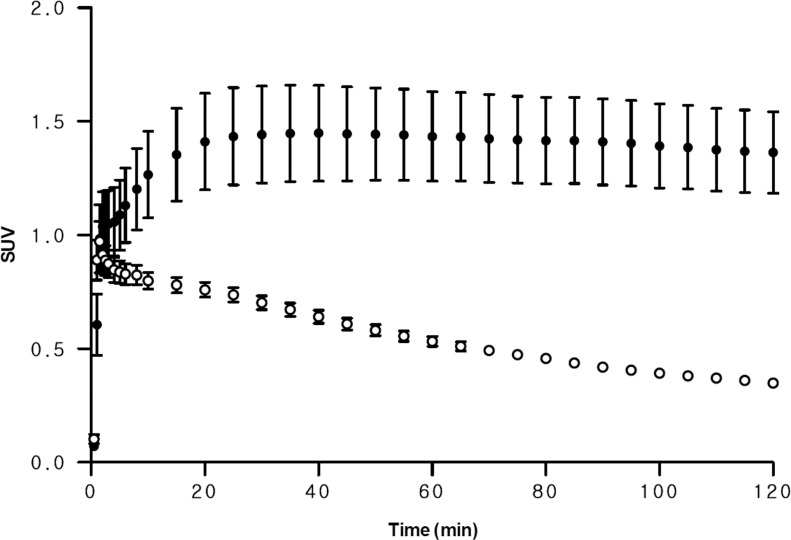
Comparison of skull uptake in ^18^F-FCWAY and ^18^F-Mefway PET. Here, ^18^F-FCWAY PET data were acquired with disulfiram pretreatment. Circles and error bars represent mean ± standard error of the mean (SEM) in five healthy controls (closed circle = ^18^F-FCWAY, open circle = ^18^F-Mefway).

The uptake of radioactivity in bone including skull is due to the presence of the radioactive ^18^F-fluoride ions resulting from metabolism of the parent compounds. The degree of radiofluorination can be used in the indicator for metabolic stability in vivo. In ^18^F-FCWAY PET, skull uptake gradually increased to 1.44 ± 0.48 SUV at 30 minutes and decreased to 1.36 ± 0.40 SUV at 120 minutes. In contrast, the skull uptake of ^18^F-Mefway rapidly reached its peak at 1.5 minutes (1.04 ± 0.21 SUV) and continuously decreased to 0.35 ± 0.02 SUV at 120 minutes (Fig. 2). At 120 minutes, brain-to-skull ratio for ^18^F-Mefway PET in the combined regions of insula and hippocampus was 1.58, which was similar to that for ^18^F-FCWAY PET (1.66).

### Comparison of regional brain uptakes

**Fig 3:**
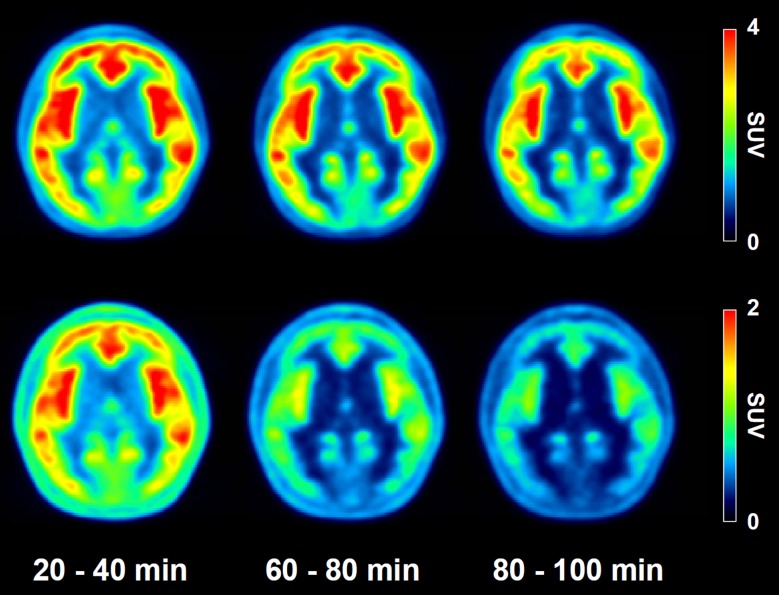
Axial PET images of ^18^F-FCWAY (upper) and ^18^F-Mefway (lower). Time-averaged images using dynamic PET images of 20–40, 60–80, and 80–100 minute, respectively.

**Fig 4:**
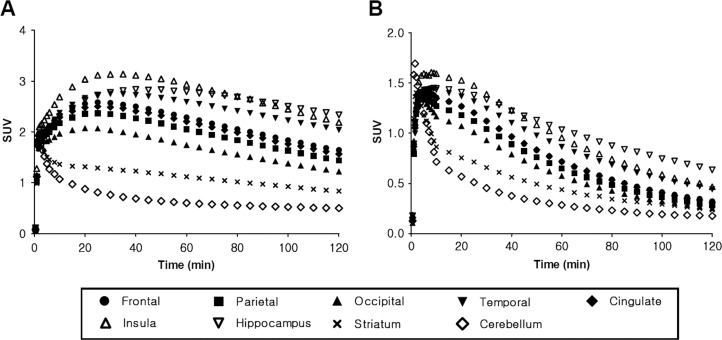
Regional time-activity curves of ^18^F-FCWAY (A) and ^18^F-Mefway (B) PET. Data represent mean values for five healthy controls.

**Fig 5:**
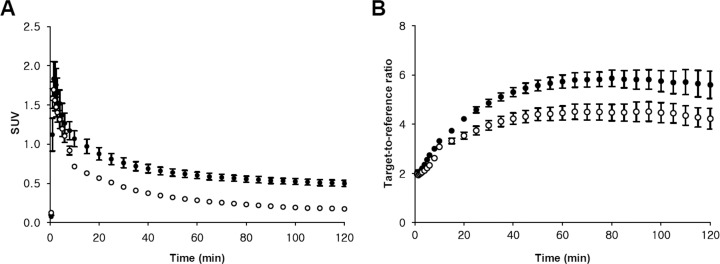
Comparison of cerebellar uptake (A) and target-to-reference ratio (B) in the hippocampus between ^18^F-FCWAY and ^18^F-Mefway PET. Circles and error bars represent mean ± standard error of the mean (SEM) in five healthy controls (closed circle = ^18^F-FCWAY, open circle = ^18^F-Mefway).

The regional uptake patterns of both radiotracers were similar: from highest to lowest in following order hippocampus, insula, temporal, frontal, cingulate, parietal, occipital cortices, striatum and cerebellum (Figs. 3 and 4). However, regional peak SUV values for the ^18^F-FCWAY PET are almost two fold than those of ^18^F-Mefway PET. In ^18^F-FCWAY PET, cortical uptake continuously increased to 3.13 SUV at 30 minutes postinjection and slowly decreased (Fig. 4A). In ^18^F-Mefway PET, the peak (1.6 SUV) appeared at 10 minutes, and the decline of radioactivity was much faster than ^18^F-FCWAY (Fig. 4B). The cerebellar radioactivity of ^18^F-FCWAY was 2.5 times higher than that of ^18^F-Mefway (Fig. 5A). Target-to-reference ratio of ^18^F-FCWAY in the combined region of insula and hippocampus is 37% higher than that of ^18^F-Mefway, and this ratio for both radiotracers reached peudo-equilibrium after 60 minutes (Fig. 5B).

### Comparison of regional binding values and parametric images

**Fig 6:**
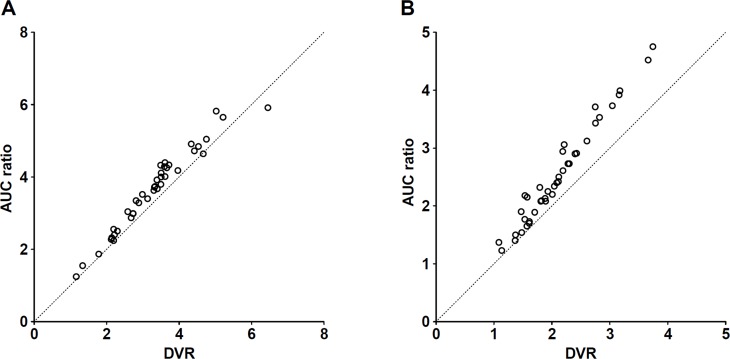
Relationship between the DVR and AUC ratio values for ^18^F-FCWAY (A) and ^18^F-Mefway (B). Dashed lines represent identity lines.

**Fig 7:**
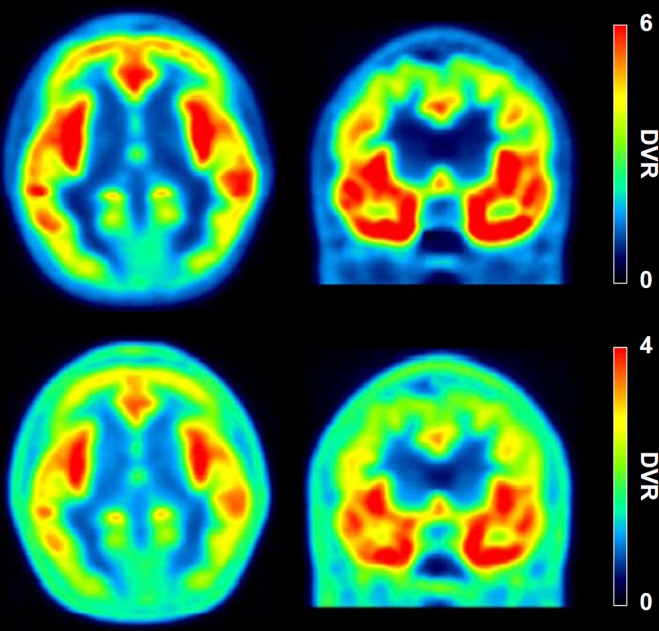
Voxel-wise parametric mapping for ^18^F-FCWAY (A) and ^18^F-Mefway (B).

Regional DVR and AUC ratio values are summarized in Table 1 and Table A in S1 File. The ^18^F-FCWAY PET showed higher DVR and AUC ratio values than ^18^F-Mefway PET by 25–63% and 23–57%, respectively. The AUC ratio values of both radiotracers strongly correlated with DVR values (R^2^ = 0.97). However, the AUC ratio values were generally overestimated compared to DVR values, and the regions with high density of 5-HT_1A_ receptor tended to show higher overestimation bias than those with low density of receptor. This overestimation bias was greater for ^18^F-Mefway than ^18^F-FCWAY (Table 1 and Fig. 6). Similarly, parametric DVR images of ^18^F-FCWAY PET showed higher DVR values than those of ^18^F-Mefway PET (Fig. 7 and Fig. A in S1 File).

**Table 1:** Regional DVR and AUC ratio values for ^18^F-FCWAY and ^18^F-Mefway PET.

Regions	^18^F-FCWAY			^18^F-Mefway		
	DVR	AUC ratio	bias	DVR	AUC ratio	bias
**Frontal**	3.27 ± 0.44	3.70 ± 0.45	0.12	2.00 ± 0.30‡	2.36 ± 0.38	0.15
**Parietal**	2.95 ± 0.48	3.32 ± 0.50	0.11	1.80 ± 0.26‡	2.11 ± 0.32	0.15
**Occipital**	2.53 ± 0.34	2.81 ± 0.34	0.10	1.63 ± 0.21‡	1.87 ± 0.25	0.13
**Temporal**	3.93 ± 0.53	4.33 ± 0.48	0.09	2.42 ± 0.39‡	2.98 ± 0.51	0.19
**Cingulate**	3.16 ± 0.63	3.62 ± 0.70	0.13	1.96 ± 0.39†	2.35 ± 0.42	0.17
**Insula**	4.32 ± 0.83	4.87 ± 0.78	0.11	2.63 ± 0.53‡	3.26 ± 0.65	0.19
**Hippocampus**	4.60 ± 1.19	4.79 ± 0.66	0.04	3.06 ± 0.68	3.82 ± 0.82	0.20
**Striatum**	1.72 ± 0.46	1.84 ± 0.40	0.07	1.35 ± 0.23	1.50 ± 0.21	0.10

Data are presented as mean ± SD. Bias was calculated as (mean AUC ratio—mean DVR)/mean AUC ratio.

*P < 0.05,

^†^P< 0.01,

^‡^P< 0.001.

## Discussion

We found that the binding pattern of ^18^F-Mefway PET was similar to that of ^18^F-FCWAY PET with disulfiram. Although lower brain uptake, lower binding values, and greater overestimation bias of AUC ratio values of ^18^F-Mefway may be shortcomings of ^18^F-Mefway compared to ^18^F-FCWAY, the greatest advantage of ^18^F-Mefway was little skull uptake resulting from in vivo defluorination. Thus, this radiotracer obviates the need for the use of disulfiram, a defluorination blocker, which may be necessary for the ^18^F-FCWAY PET in order to obtain clean brain signals. In addition, the radioactivity in the cerebellum for the ^18^F-Mefway PET was lower than that of ^18^F-FCWAY. Cerebellum is regarded as non-specific binding region because this area is almost devoid of 5-HT_1A_ receptors. The low cerebellar uptake gives rise to high contrast PET images results from the low background. This result is similar to the binding data in Table 2. Binding affinity of ^18^F-Mefway showed the highest value, and the binding potential of ^18^F-Mefway was almost two fold higher than the ^18^F-MPPF, same ^18^F-labeled radioligand for 5-HT_1A_ receptor [28]. Therefore, ^18^F-Mefway may be a promising radioligand for 5-HT_1A_ receptor imaging in human.

**Table 2:** Comparison of the radiotracers for WAY-100635 derivatives.

Radiotracer	Binding affinity^a^(IC_50_, nM)	Binding potential				
		Frontal	Temporal	Insula	Cingulate	Hippocampus
^11^C-WAY-100635^b^	-	3.23	4.75	5.45	4.2	4.24
^18^F-MPPF^c^	3.66	0.61	0.85	0.96	0.75	1.43
^18^F-FCWAY	2.16	2.27	2.93	3.32	2.16	3.60
^18^F-Mefway	1.49	1.11	1.50	1.75	1.09	2.07

^a^IC_50_ measured in human recombinant 5-HT_1A_ receptors expressed in Chinese hamster ovary cells by using ^3^H-WAY-100635 [28].

^b, c^ Binding potential values for ^11^C-WAY-100635 and ^18^F-MPPF are calculated differently [37], [38].

For ^18^F-FCWAY and ^18^F-Mefway, binding potential values were calculated as DVR—1. DVR was a mean value in the various brain regions (this work).

Reference method for kinetic modeling utilizes indirect input curves in the reference tissue which is devoid of interesting receptors. Cerebellar gray matter and vermis contain noticeable 5-HT_1A_ receptors whereas cerebellar white matter has few 5-HT_1A_ receptors in humans [25]. Therefore we calculated regional binding values using the white matter as previously recommended [29,30]

In the present study, the brain uptake values of ^18^F-Mefway PET were lower than those estimated in the rhesus monkey [31]. This discrepancy may be partly explained by the species differences in the enzyme system metabolizing the radiotracer and the substrate activity for P-gp. First, cytochrome P450 (CYP) transforms lipophilic drugs into more polar compounds that can be easily eliminated from the body [32]. The majority of CYP enzymes are conserved among species but some isoforms of CYP were shown to generate species differences in drug metabolism [33]. For instance, various CYP3A isoforms exist in difference species. CYP3A4, 3A5, 3A7 and 3A43 are the major drug-metabolizing isoforms for humans whereas CYP3A8 is important for monkey. Second, P-gp is an efflux transporter located in the apical side of the endothelial cell of the blood brain barrier (BBB) and plays a role in removal of potentially harmful compounds such as toxic substances from the brain [34]. Similarly, the brain uptake of a radiotracer, which is a substrate for P-gp, can be affected by P-gp function. The ^11^C-(R)-RWAY and ^18^F-MPPF, 5-HT_1A_ receptor antagonists, were found to be P-gp substrates in rodents and showed low brain uptake [12,35]. Thus, it may be possible that the P-gp function partially contributed to lower brain radioactivity of ^18^F-Mefway PET in this study. However, the intervention of different CYP enzymes and P-gp merits further investigation.

Faster plasma clearance and slower tissue clearance increases the apparent volume of distribution and target-to-reference ratio in transient equilibrium, and thus, this results in overestimation bias compared to the total volume of distribution and DVR values derived from kinetic modeling. Moreover, the overestimation bias is clearly affected by regional receptor density [36]. In the present study, the AUC ratio values were higher than the DVR values for both radiotracers, and the overestimation bias of AUC ratio values were higher for the ^18^F-Mefway than ^18^F-FCWAY. The regional overestimation bias of ^18^F-Mefway was disproportional; highest in the hippocampus (20%) and lowest in the striatum (10%). AUC tissue to reference ratio can easily applied to estimate the receptor binding to the target tissue. This large bias deduces low accuracy of AUC ratio methods, therefore care should be taken for the large clinical studies of ^18^F-Mefway PET with static PET scans.

Although defluorination and resulting high skull uptake is a major metabolic problem for some ^18^F-labeled radioligands, there is no uniform, established method to prevent this issue. The CYP2E1 and glutathione S-transferase are related with the defluorination of ^18^F-FCWAY and ^18^F-SP203, respectively. Disulfiram, a direct CYP2E1 inhibitor, can be applied to prevent defluorination in human [24]. Because of its known neurotoxicity particularly to the globus pallidus, striatum, and substantia nigra and resulting extrapyramidal signs, its clinical application may be limited [13,14]. Thus, little skull uptake ^18^F-Mefway even without defluorination blocker is greatest advantage over ^18^F-FCWAY.

There are limitations in this study. First, we did not acquire plasma input function nor metabolite assay, which might useful for fully analyzing the characteristics of ^18^F-Mefway. Second, we did not correct the cerebellum for vascular radioactivity or uptake of labeled mebolites as has been done with previous ^18^F-FCWAY studies. These factors may be causes for a moderate decrease in target-to-reference ratios at the late times. Further studies should be necessary to make these things clear.

## Conclusion

Although the ^18^F-Mefway PET showed relatively low brain uptake and DVR values compared to ^18^F-FCWAY PET with disulfiram pretreatment, ^18^F-Mefway has reasonable binding values with little skull uptake. Therefore, ^18^F-Mefway may be a good candidate PET radioligand in human use.

## Supporting Information

S1 File Fig. A, Comparison of voxel-wise parametric mapping for 18F-FCWAY (A) and 18F-Mefway (B). Table A, Comparison of reference tissue models.
(DOCX)
