# Correction: ^18^F-Mefway PET Imaging of Serotonin 1A Receptors in Humans: A Comparison with ^18^F-FCWAY

**DOI:** 10.1371/journal.pone.0127491

**Published:** 2015-04-27

**Authors:** Jae Yong Choi, Chul Hyoung Lyoo, Jin Su Kim, Kyeong Min Kim, Jee Hae Kang, Soo-Hee Choi, Jae-Jin Kim, Young Hoon Ryu

In the Funding section, the grant number from the funder Yonsei University College of Medicine is listed incorrectly. The correct grant number is: 6-2008-0237.
