# Supplementary material for: 18F-Mefway PET Imaging of Serotonin 1A Receptors in Humans: A Comparison with 18F-FCWAY
Source: PLoS One. 2015 Apr 1;10(4):e0121342. doi: 10.1371/journal.pone.0121342 (PMC4382022; doi:10.1371/journal.pone.0121342)
Supplement: S1 File — Table A, Comparison of reference tissue models. (DOCX) [file pone.0121342.s001.docx]

**Supporting Information**

**Table A in S1** Comparison of reference tissue models

| **Regions** | **^18^F-FCWAY** | | |  | **^18^F-Mefway** | | |
| --- | --- | --- | --- | --- | --- | --- | --- |
|  | **SRTM** | **MRTM2** | **Ref. Logan** |  | **SRTM** | **MRTM2** | **Ref. Logan** |
| **Frontal** | 3.44 ± 0.39 | 3.36 ± 0.44 | 3.27 ± 0.44 |  | 2.06 ± 0.28 | 2.03 ± 0.30 | 2.00 ± 0.30 |
| **Parietal** | 3.10 ± 0.44 | 3.02 ± 0.48 | 2.95 ± 0.48 |  | 1.86 ± 0.24 | 1.82 ± 0.26 | 1.80 ± 0.26 |
| **Occipital** | 2.65 ± 0.29 | 2.58 ± 0.33 | 2.53 ± 0.34 |  | 1.69 ± 0.19 | 1.66 ± 0.20 | 1.63 ± 0.21 |
| **Temporal** | 3.99 ± 0.44 | 4.00 ± 0.55 | 3.93 ± 0.53 |  | 2.41 ± 0.34 | 2.43 ± 0.38 | 2.42 ± 0.39 |
| **Cingulate** | 3.34 ± 0.61 | 3.27 ± 0.67 | 3.16 ± 0.63 |  | 2.02 ± 0.37 | 1.98 ± 0.38 | 1.96 ± 0.39 |
| **Insula** | 4.47 ± 0.72 | 4.41 ± 0.85 | 4.32 ± 0.83 |  | 2.65 ± 0.47 | 2.65 ± 0.53 | 2.63 ± 0.53 |
| **Hippocampus** | 4.48 ± 0.76 | 4.58 ± 1.06 | 4.60 ± 1.19 |  | 2.93 ± 0.58 | 3.06 ± 0.67 | 3.06 ± 0.68 |
| **Striatum** | 1.75 ± 0.40 | 1.74 ± 0.44 | 1.72 ± 0.46 |  | 1.32 ± 0.21 | 1.35 ± 0.21 | 1.35 ± 0.23 |


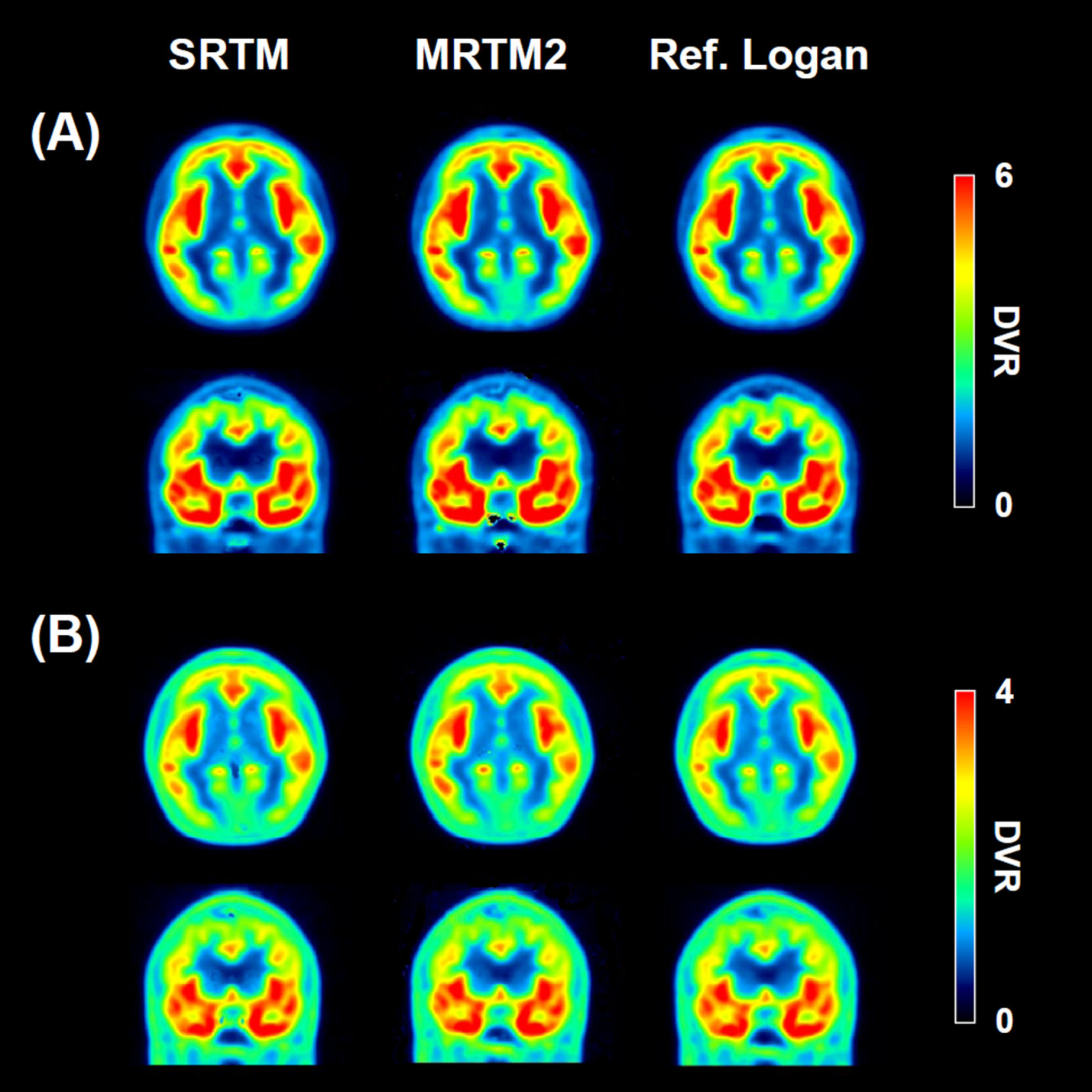


**Fig A in S1.** Comparison of voxel-wise parametric mapping for ^18^F-FCWAY (A) and ^18^F-Mefway (B)
